# Supplementary material for: Quantifying how constraints limit the diversity of viable routes to adaptation
Source: PLoS Genet. 2018 Oct 8;14(10):e1007717. doi: 10.1371/journal.pgen.1007717 (PMC6193742; doi:10.1371/journal.pgen.1007717)
Supplement: S1 Data — (DOCX) [file pgen.1007717.s001.docx]

Supplementary Materials

Source code providing functions for calculating all metrics discussed in this paper can be found at https://github.com/samyeaman/dgconstraint.

**Quantifying diversity constraints in pairwise contrasts: Set notation**

We use the following set notation to represent various aspects of how these lineages are adapted: the sets of genes in their genomes are *N_x_* and *N_y_*, with the subset of genes common to both lineages being *N_s_* = *N_x_* ∩ *N_y_*; the adapted trait is controlled by *G_x_* and *G_y_* genetic loci in each lineage; of the *G_x_* and *G_y_* loci that could potentially give rise to variation in the trait, only subsets of these loci, *GA_x_* and *GA_y_*, are likely to contribute to variation; the sets of genes that are locally adapted within each lineage are *A_x_* and *A_y_*. The size of each of these sets (*i.e*., set cardinality) is represented with the corresponding lowercase letter (*a_s_* = |*A_s_*|, *n_x_* = |*N_x_*|, *etc*.), and it follows that the locally adapted genes are a subset of the genes making up the mutational target, which are a subset of the total set of genes in the genome (*A* ⊂ *G* ⊂ *N*).

We can partition the members of the set *G_j_* into orthologs that are shared by both lineages and are members of set *N_s_* ($G_{j}^{'}$) and genes that are only present in lineage *j* ($G_{j}^{*}$). For simplicity, we begin under the assumption that all genes that potentially drive variation in the trait (*G_x_* and *G_y_*) are members of *N_s_*, so that $G_{x}^{'}=G_{x}$ and $G_{y}^{'}=G_{y}$ and $G_{x}^{*}=\emptyset$ and $G_{y}^{*}=\emptyset$ (empty set). Under this assumption, the set of genes that potentially give rise to variation in both lineages is *G_s_* = *G_x_* ∩ *G_y_*, and the set of genes that potentially contribute to adaptation in both lineages is *GA_s_*. Similarly, we can partition the genes that are locally adapted within a given lineage (*A_j_*) into those that make up the mutational target in both lineages and are members of set *G_s_* ($A_{j}^{'}$) and those that only potentially contribute to variation within lineage *j* ($A_{j}^{*}$). Again, for simplicity we begin under the assumption that all locally adapted genes (*A_x_* and *A_y_*) are members of *G_s_*, so that $A_{x}^{'}=A_{x}$ and $A_{y}^{'}=A_{y}$ and $A_{x}^{*}=\emptyset$ and $A_{y}^{*}=\emptyset$. Under this assumption, the set of genes that are locally adapted within both lineages (i.e. convergently evolved) is therefore *A_s_* = *A_x_* ∩ *A_y_* (Figure S1).

Figure S1: Schematic representation of the overlap between the genes that potentially contribute to variation (*G*) and adaptation (*GA*) and those observed to contribute to adaptation (*A*) within two lineages (*x* and *y*) undergoing local adaptation to a similar selection pressure. Note that for simplicity *N_x_* and *N_y_* are not shown, and this figure is drawn under the assumption that all members of *G_x_* and *G_y_* are also members of *N_s_*, and all members of *GA_x_* and *GA_y_* are also members of *G_x_* and *G_y_* (*i.e.*, $G_{x}^{'}=G_{x}$ and $G_{y}^{'}=G_{y}$ and $G_{x}^{*}=\emptyset$ and $G_{y}^{*}=\emptyset$)*.*

**Deviation between Pearson’s and analytical *χ^2^***

If we consider the simplest scenario where all genes have equal probabilities of contributing to adaptation in all lineages, then$e=\sum_{i} o_{i}/g_{0}$. Using binomial probabilities to simulate a distribution of ***χ^2^*** statistics, setting higher values of *e_j_* results in greater discrepancy between the observed mean of ***χ^2^*** and the analytical prediction that E[***χ^2^***] = *df* (Figure S2A). Similarly, the relative deviation between the mean of Pearson’s ***χ^2^*** and the analytical ***χ^2^*** statistics increases with decreasing degrees of freedom (Figure S2B; in this case *df* = *g*_0_ - 1). Thus, standardizing observed test statistics by the degrees of freedom or the total number of mutations will not accurately represent the deviation from expectations across widely varying scenarios.

Figure S2: Deviations between the distribution of Pearson’s statistic for *χ^2^*  and the exact *χ^2^*  distribution for the same degrees of freedom, for simulations with varying numbers of genes with adaptive mutations per replicate (A), and varying numbers of genes underlying the trait (B). Solid vertical bars show analytical means, dashed vertical lines show simulated means. In all cases *k* = 10; in panel A, *g_s_* = 100; in panel B, *a_j_* = 50.

**Comparison of hypergeometric vs likelihood quantification of *ga*_s_**

Both methods for estimating the number of genes that actually have the potential to contribute to adaptation (*ga*_s_; rather than just to standing variation, *g_s_*) have limitations that affect the accuracy of inferences. For the hypergeometric approach where $\hat{ga}_{s}=a_{x}a_{y}/a_{s}$, the estimator becomes undefined if *a_s_* = 0 in any pairwise comparison, so in such cases it is necessary to either set *ga*_s_ *= g_s_* (or *n_s_*) for the pairwise contrast or to simply not use the test. By contrast, this is not a limitation for the likelihood-based method for estimating *ga*_s_. Both tests assume that all *ga*_s_ loci have equal probability of contributing to adaptation, which is biologically unlikely and will lead to bias. The difficulties associated with this can be illustrated by contrasting two examples: in scenario 1 there are *k* lineages and each lineage is adapted via a different gene, so that *a_s_* = 0 for all pairwise contrasts (effectively no constraints); scenario 2 has the same pattern but one of the *k* genes is also adapted in all lineages (no constraints except adaptation seems to require the one highly repeatable gene). In both scenarios, we would expect the number of genes that are actually contributing to adaptation over all lineages would scale with the number of lineages included in the study, so intuitively we would infer that all genes have the potential to contribute to adaptation and *ga*_s_ *= g_s_* = *n_s_*. However, if there are *g_s_* = *n_s_* = 5000 genes in the genome, the likelihood based estimator would infer $\hat{ga}_{s}=4999.5$ for scenario 1 (consistent with intuition), but $\hat{ga}_{s}=25$ for scenario 2 (under *k* = 20 lineages). Similarly, the hypergeometric-based estimation would yield $\hat{ga}_{s}=5000$ for scenario 1 (consistent with intuition), but $\hat{ga}_{s}=3.8$ for scenario 2 (under *k* = 20 lineages).

**Comparison of *C*-scores to Jaccard and PS-metrics**

To explore the relationship between *C_chisq_* and *C_hyper_* and compare them to the other metrics across a broader range of scenarios, we simulated datasets with different numbers of genes (*g*_0_ = *g_s_*) by randomly drawing a given number of adapted genes (*a_i_*) in each lineage. We then created a range of scenarios with increasingly convergent patterns by sorting an increasing fraction of the rows in each lineage, as this maximizes the overlap in the sorted rows. Across these simulated datasets, the *C_chisq_* statistic varies linearly with the *C_hyper_* (Figure S3), with the upper bound increasing as a function of the number of genes, which is a desirable property for representing the amount of constraint, as observing a given number of genes overlapping becomes increasingly unlikely as the *g_s_* increases. By contrast, the Jaccard and PS*_add_* yield the same measure of convergence for scenarios with very different probabilities of this occurring by chance, and they are largely non-congruent with the exact probability-based estimates of *C_hyper_* (Figure S3). This is because they represent how often the same genes are used repeatedly in adaptation rather than quantify the probability of deviation from expectations caused by constraints.

**Repeatability in simulations with a variable distribution of mutation effect sizes**

In addition to the simulations shown in Figure 5, we also ran simulations with a constant size of mutational target and variable distribution of allele effect size across loci.

In this case, simulations had *n* = *g_s_* = 100 causal loci with allele effect sizes drawn from a gamma distribution with mean = 0.02 and variable shape parameters. At small values of the shape parameter most alleles have small effect sizes and only a few have very large sizes, while at high values of the shape parameter allele effect sizes are nearly uniform (effect sizes were truncated to a minimum of 0.00001).

Under very small values of the gamma shape parameter, the distribution of allele effect sizes tends to be made up of many small effect alleles and a few large-effect ones, which means that sometimes there are not suitable combinations of alleles that can combine to yield a locally optimal phenotypic value (Figure S4). The repeatability is then strongly dependent on the particular combination of allele effect sizes drawn for the simulation run, and some cases have low repeatability (Figure S4B&C) while others have high repeatability (Figure S4D&E). With intermediate values of the shape parameter, there are a smaller number of loci with alleles of intermediate effect size and evolution becomes highly repeatable across a larger number of loci, yielding an increase in *C_chisq_* (Figure S4F&G). At high values of the shape parameter, all loci have small effect sizes close to the mean and are therefore nearly interchangeable in their fitness effects (Figure S4H&I). Also, because the net effect of selection is smaller relative to migration these alleles fall much closer to the swamping threshold, so in many cases *F_ST_* is not sufficiently elevated for adaptation to be detectable based on the criteria used here (*i.e*., motif II in Yeaman 2015). As a result, at high values of the shape parameter there is very little overlap in the loci that contribute to adaptation and very low values of *C_chisq_*. The continuous metric of *C_chisq_* is much less noisy than the binary metric at high values of the gamma shape parameter, as a result of the arbitrary cut-off used for categorizing adaptation based on *F_ST_*.

Figure S3. Comparison of *C_hyper_* with *C_chisq_* (A, B), Jaccard (C, D), and PS*_add_* metrics (E, F) for quantifying convergence under scenarios with different numbers of adaptive mutations (A-C) and causal loci (D-F). In all cases, scenarios were simulated for two replicate lineages with *a_i_* adaptive mutations in one and *a_i_* + 20 adaptive mutations in the other. In panels A,C & E *g_s_* = 200; in panels B, D & F *a_i_* = 10. For each parameter set, a number of simulations were run, each with a different proportion of the rows in each lineage sorted numerically, to introduce different amounts of repeatability into each run (*i.e*., the same procedure as in Figure 3).

Figure S4. The metrics of convergence in individual based simulations exhibit a non-monotonic pattern with increasing values of the gamma shape parameter, which changes GF-redundancy (panel A). Example plots show divergence at individual loci contributing to local adaptation for four of the replicate simulation runs plotted in the left-hand panel. Panels B, D, F, & H show binned *F_ST_* values for each locus, averaged over the last 25 census points; panels C, E, G & I show histograms of the allele effect sizes in the simulation.
