# Supplementary figures and images for: Quantifying how constraints limit the diversity of viable routes to adaptation
Source: PLoS Genet. 2018 Oct 8;14(10):e1007717. doi: 10.1371/journal.pgen.1007717 (PMC6193742; doi:10.1371/journal.pgen.1007717)

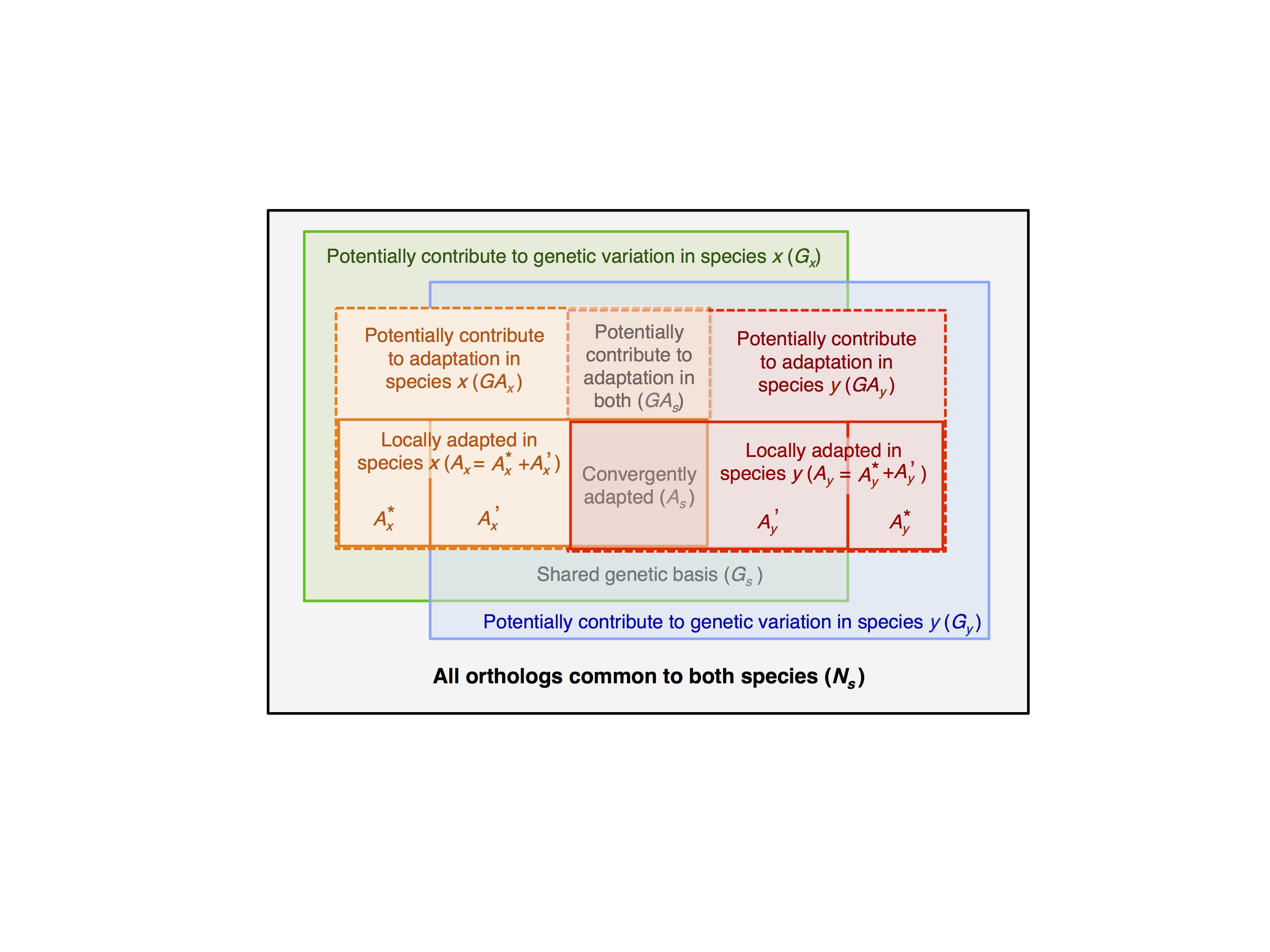

Supplement: S1 Fig — Note that for simplicity Nx and Ny are not shown, and this figure is drawn under the assumption that all members of Gx and Gy are also members of Ns, and all members of GAx and GAy are also members of Gx and Gy (i.e., Gx'=Gx and Gy'=Gy and Gx*=∅ and Gy*=∅). (TIFF) [file pgen.1007717.s002.tiff]

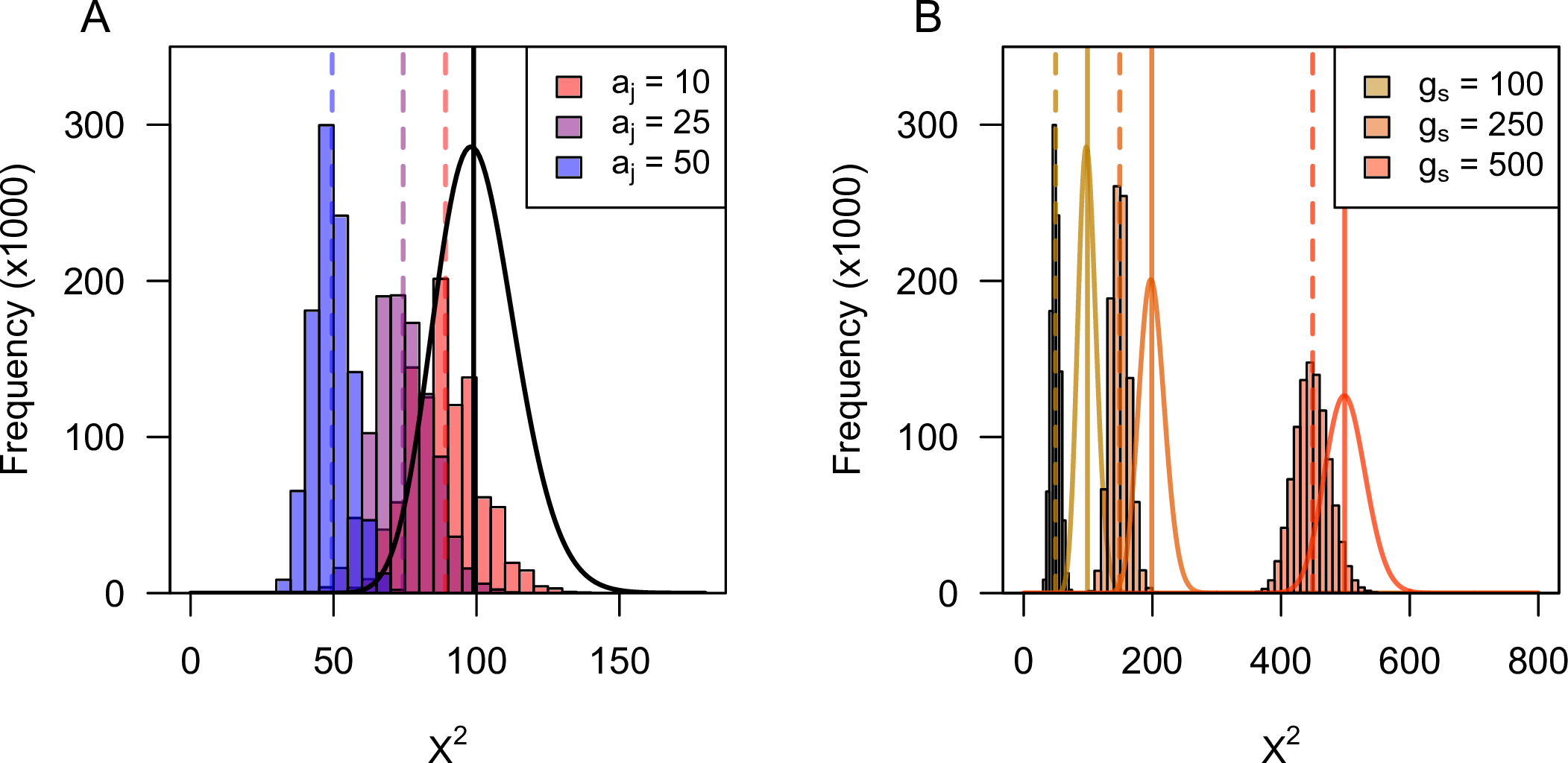

Supplement: S2 Fig — Solid vertical bars show analytical means, dashed vertical lines show simulated means. In all cases k = 10; in panel A, gs = 100; in panel B, ai = 10. (TIF) [file pgen.1007717.s003.tif]

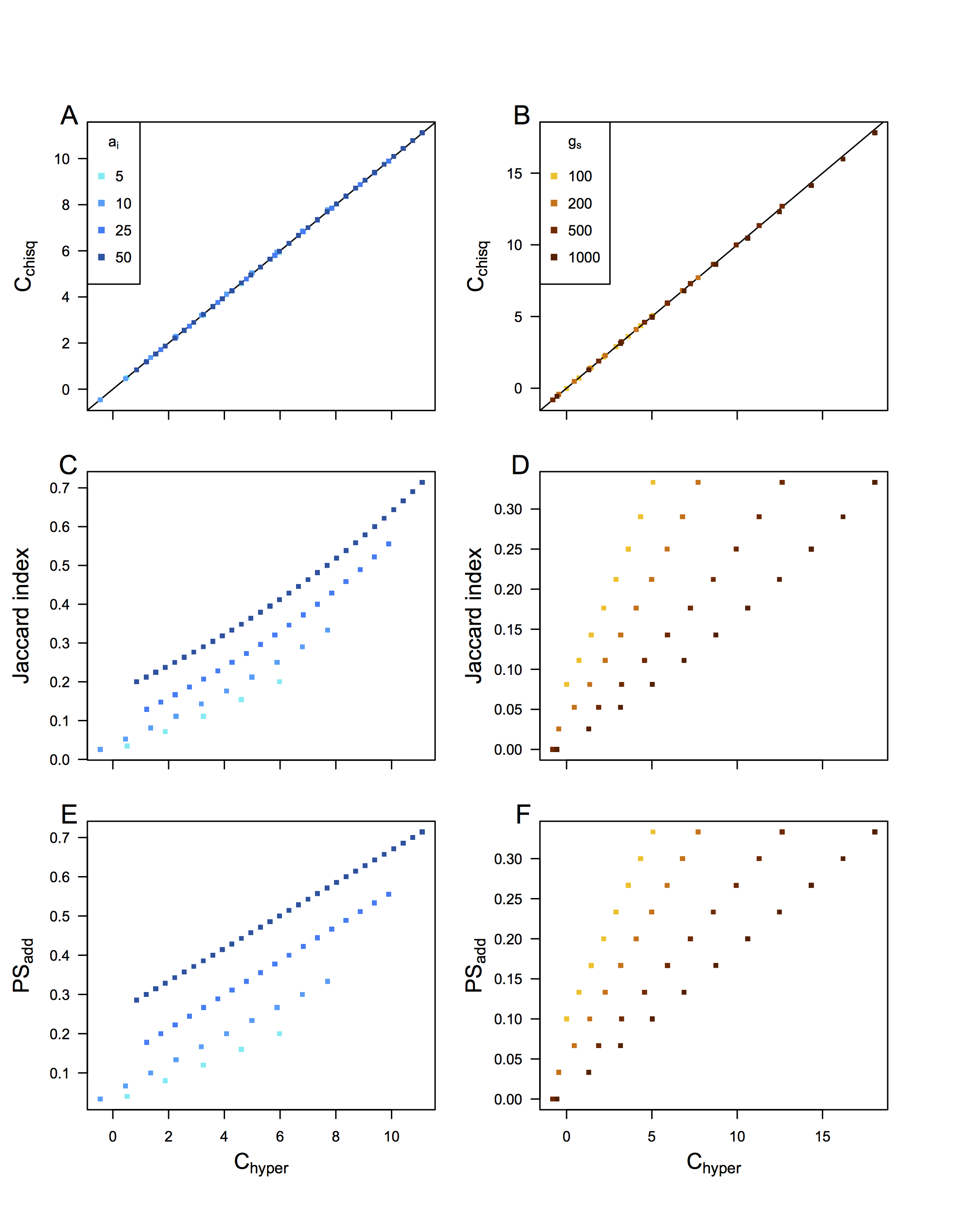

Supplement: S3 Fig — In all cases, scenarios were simulated for two replicate lineages with ai adaptive mutations in one and ai + 20 adaptive mutations in the other. In panels A, C & E gs = 200; in panels B, D & F ai = 10. For each parameter set, a number of simulations were run, each with a different proportion of the rows in each lineage sorted numerically, to introduce different amounts of repeatability into each run (i.e., the same procedure as in Fig 3). (TIFF) [file pgen.1007717.s004.tiff]

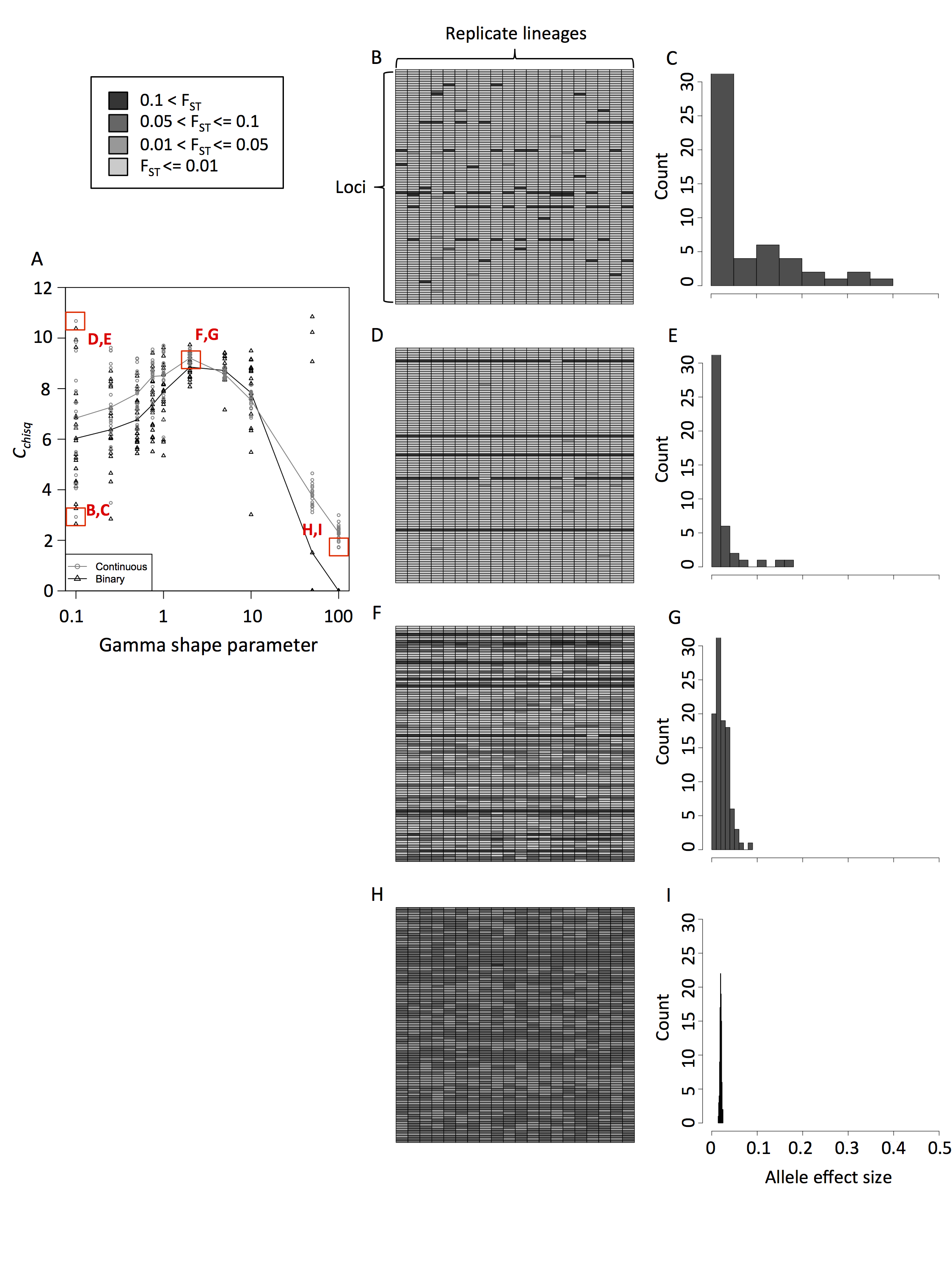

Supplement: S4 Fig — Example plots show divergence at individual loci contributing to local adaptation for four of the replicate simulation runs plotted in the left-hand panel. Panels B, D, F, & H show binned FST values for each locus, averaged over the last 25 census points; panels C, E, G & I show histograms of the allele effect sizes in the simulation. (TIFF) [file pgen.1007717.s005.tiff]
